# Acylated Anthocyanins from Red Cabbage and Purple Sweet Potato Can Bind Metal Ions and Produce Stable Blue Colors

**DOI:** 10.3390/ijms22094551

**Published:** 2021-04-27

**Authors:** Julie-Anne Fenger, Gregory T. Sigurdson, Rebecca J. Robbins, Thomas M. Collins, M. Mónica Giusti, Olivier Dangles

**Affiliations:** 1 Avignon University, INRAE, UMR408, 84000 Avignon, France; 2 Department of Food Science and Technology, The Ohio State University, 2015 Fyffe Ct., Columbus, OH 43210, USA; sigurdson.g@gmail.com (G.T.S.); giusti.6@osu.edu (M.M.G.); 3 Mars Wrigley, 1132 W Blackhawk Street, Chicago, IL 60642, USA; rebecca.robbins@effem.com (R.J.R.); daddoc2013@gmail.com (T.M.C.)

**Keywords:** anthocyanin, hydroxycinnamic acid, polyphenol, pigment, acylation, aluminum, iron, metal complexation

## Abstract

Red cabbage (RC) and purple sweet potato (PSP) are naturally rich in acylated cyanidin glycosides that can bind metal ions and develop intramolecular π-stacking interactions between the cyanidin chromophore and the phenolic acyl residues. In this work, a large set of RC and PSP anthocyanins was investigated for its coloring properties in the presence of iron and aluminum ions. Although relatively modest, the structural differences between RC and PSP anthocyanins, i.e., the acylation site at the external glucose of the sophorosyl moiety (C2-OH for RC vs. C6-OH for PSP) and the presence of coordinating acyl groups (caffeoyl) in PSP anthocyanins only, made a large difference in the color expressed by their metal complexes. For instance, the Al^3+^-induced bathochromic shifts for RC anthocyanins reached ca. 50 nm at pH 6 and pH 7, vs. at best ca. 20 nm for PSP anthocyanins. With Fe^2+^ (quickly oxidized to Fe^3+^ in the complexes), the bathochromic shifts for RC anthocyanins were higher, i.e., up to ca. 90 nm at pH 7 and 110 nm at pH 5.7. A kinetic analysis at different metal/ligand molar ratios combined with an investigation by high-resolution mass spectrometry suggested the formation of metal–anthocyanin complexes of 1:1, 1:2, and 1:3 stoichiometries. Contrary to predictions based on steric hindrance, acylation by noncoordinating acyl residues favored metal binding and resulted in complexes having much higher molar absorption coefficients. Moreover, the competition between metal binding and water addition to the free ligands (leading to colorless forms) was less severe, although very dependent on the acylation site(s). Overall, anthocyanins from purple sweet potato, and even more from red cabbage, have a strong potential for development as food colorants expressing red to blue hues depending on pH and metal ion.

## 1. Introduction

The color of red cabbage (RC) and purple sweet potato (PSP) is due to closely related anthocyanins displaying a cyanidin or peonidin (3′-O-methylcyanidin) 3-O-sophoroside-5-O-glucoside structure [1,2]. Cyanidin derivatives (the major RC pigments) are especially interesting colorants owing to their ability to bind metal ions (via their catechol B-ring) in neutral or mildly acidic solution. Indeed, metal-induced cyanidin deprotonation leads to a quinonoid chromophore that can express intense purple to blue colors [1,3]. Another important consequence of metal–anthocyanin binding is increased color stability. Indeed, through the electrophilic flavylium ion (main colored form in acidic solution), anthocyanins, unlike their metal chelates, are vulnerable to water addition with the concomitant reversible formation of colorless forms (hemiketal and chalcones) [4]. A remarkable feature of RC anthocyanins is that the sophorosyl moiety is typically acylated by one or two residue(s) of *p*-hydroxycinnamic acid (HCA = *p*-coumaric, ferulic, caffeic, or sinapic acid). Phenolic acyl groups are known to favor folded conformations in which the anthocyanidin (chromophore) and the acyl residues develop π-stacking interactions. Like metal binding, this phenomenon, called intramolecular copigmentation, causes a bathochromic shift (BS) in the visible absorption band and protects the chromophore against water addition [5,6,7]. The combination of π-stacking interactions and metal binding is actually required to achieve maximal blue color stability with anthocyanins. Indeed, phenolic acyl groups stacked onto the cyanidin nucleus could either directly participate in metal binding (e.g., caffeic acid residues through their catechol ring) or at least strengthen metal binding by building a hydrophobic pocket around the metal–cyanidin complex. Recently, a remarkable RC anthocyanin (called pigment B or PB), displaying a single ideally located sinapoyl residue, was shown to form an aluminum(III) complex of 1:3 stoichiometry in which the three PB ligands in octahedral coordination to Al^3+^ adopt a chiral arrangement, causing an intense positive Cotton effect in the visible part of its circular dichroism spectrum [8]. The Al(PB)_3_ complex is strongly stabilized by the π-stacking interactions taking place between each cyanidin chromophore and the sinapoyl residue of an adjacent ligand. Moreover, this original supramolecular structure imposes a large torsion angle around the bond connecting the B- and C-rings of the three cyanidin nuclei. This unique combination of structural characteristics results in an intense vibrant blue color of high stability, making PB and its metal complexes potential lead compounds for the replacement of artificial blue colorants by natural alternatives.

In this work, a selection of acylated cyanidin glycosides from red cabbage (including PB) and purple sweet potato is revisited for its affinity for aluminum and iron ions. In particular, the influence of the acyl groups on the binding kinetics, color stability, and rate of oxidative degradation of the complexes is systematically addressed. Indeed, the addition of iron ions was shown to accelerate the oxidative degradation of nonacylated cyanidin glycosides, while the presence of phenolic acyl groups tends to cancel this effect [9]. In this work, the conditions (pH, metal type, and concentration) permitting the optimal development of a blue color is also explored.

## 2. Results and Discussion

Metal–anthocyanin binding is of great importance for plants, not only because it is an efficient way to express blue colors to attract pollinating insects [6], but also as a detoxification mechanism against metal excess [10], which can operate with a variety of metal ions (e.g., Fe, Al, Pb, Cd, Mo, Mg, Ni, and V in corn roots). With Cu^2+^, the binding is followed by Cu^2+^ reduction and anthocyanin oxidation [11]. However, quantitative physicochemical investigations of metal–anthocyanin binding are scarce. Such approaches have to address the structural transformations of anthocyanins in aqueous solution, the kinetics of metal binding and its stoichiometry, the critical influence of pH, and the possible influence of phenolic acyl groups and even of the selected buffer (depending on its own affinity for metal ions). This is the specific focus of this work, based on a large series of diversely acylated cyanidin glycosides and two of the most important metals (Al, Fe) in terms of blue color development [6].

### 2.1. The Color and Spectral Properties of the Metal Complexes

**Figure 1:**
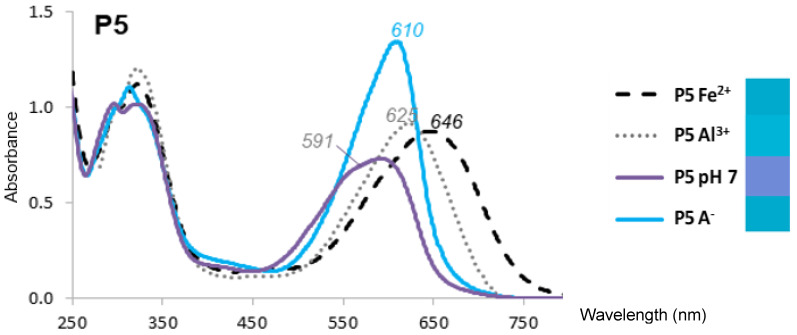
UV-VIS spectra of Pigment 5 (50 µM) at pH 7, its pure anionic base (A^−^, calculated), and its Fe^2+^ and Al^3+^ complexes (1 equiv.). Right: color patches from the L*a*b* coordinates calculated from the visible spectra.

Anthocyanins under their flavylium form (AH^+^) are typically diacids undergoing a first proton loss from C7-OH (p*K*_a1_ ≈ 4), followed by a second one from C4′-OH around neutrality [12]. Thus, at pH 7, cyanidin glycosides from RC or PSP are a mixture of neutral (A_7_) and anionic (A_4′7_) bases (p*K*_a2_ = 7.0–7.3 [7,13]), in agreement with the broad absorption band observed (Figure 1). From the experimental spectra at pH 5–7, the p*K*_a_ values, and the spectrum of the pure flavylium ion (pH 1), the spectra of the pure neutral and anionic bases can be calculated [13]. Compared to the neutral base, the anionic base not only has a much higher *λ*_max_, but also a higher molar absorption coefficient at *λ*_max_ (Figure 1).

**Scheme 1:**
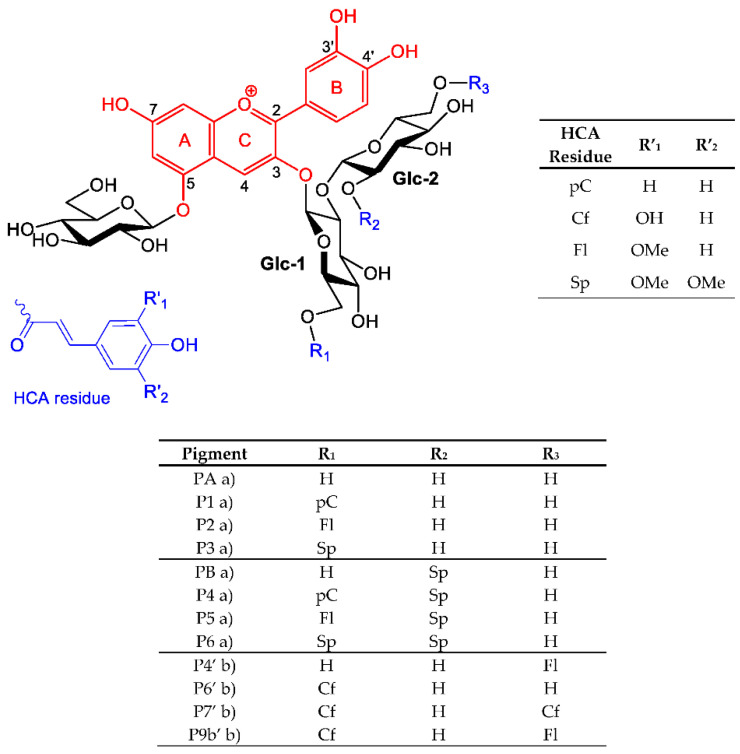
Structure of the RC and PSP anthocyanins studied. When present, hydroxycinnamoyl residues R_1_, R_2_, and R_3_ are *p*-coumaroyl (pC), feruloyl (Fl), caffeoyl (Cf), and/or sinapoyl (Sp). Note: a) from red cabbage; b) from purple sweet potato.

The major PSP anthocyanins are acylated peonidin glycosides, which do not bind metal ions through their chromophore. However, cyanidin glycosides are also present in PSP. Unlike the RC anthocyanins, the HCA residues of the PSP anthocyanins are only located at the primary C6-OH positions of the sophorosyl moiety (Scheme 1). In particular, the acyl residue borne by Glc-2 (R_3_) is expected to be more mobile than its homolog in red cabbage (R_2_ = sinapoyl). This happens to make a large difference in terms of color variation: the Al^3+^-induced bathochromic shifts for PB (red cabbage) are ca. 50 nm at pH 6 and pH 7 vs. at best ca. 20 nm for P4′ (R_3_ = feruloyl, Table 1). The flexible acyl residue of P4′ is probably much less apt to develop π-stacking interactions with the cyanidin nucleus than the more rigid sinapoyl residue of PB. Consistently, it has been demonstrated that RC anthocyanins having a single HCA residue at R_1_ (P1–P3) are much more susceptible to water addition than PB [8] as a consequence of the latter adopting folded conformations in which the cyanidin and HCA moieties are in molecular contact. However, PSP pigments have a specific advantage over RC pigments: the presence of caffeoyl residues at Glc-1 and/or Glc-2, which themselves can bind metal ions.

Our recent work [13] suggests that such pigments, in which the cyanidin and caffeoyl units tend to stack onto each other, can actually sequester Fe^2+^ and Al^3+^ by the simultaneous involvement of both catechol rings, thereby largely increasing the metal-induced bathochromic shift. This was confirmed in the current work: the bathochromic shifts induced by Al^3+^ (1 equiv.) at pH 7 were 8, 21, and 22 nm for P4′ (R_3_ = feruloyl), P6′ (R_1_ = caffeoyl), and P7 (R_1_ = R_3_ = caffeoyl), respectively (Figure S1A, Table 1). It was also clear that the caffeoyl residue at R_1_ was critical to promote the bluing effect, while the other one at R_3_ was not. By contrast, P9b (R_1_ = caffeoyl, R_3_ = feruloyl) was clearly less efficient (BS = 16 nm), possibly pointing to a less-favorable binding because of the relatively bulky feruloyl residue.

**Scheme 2:**
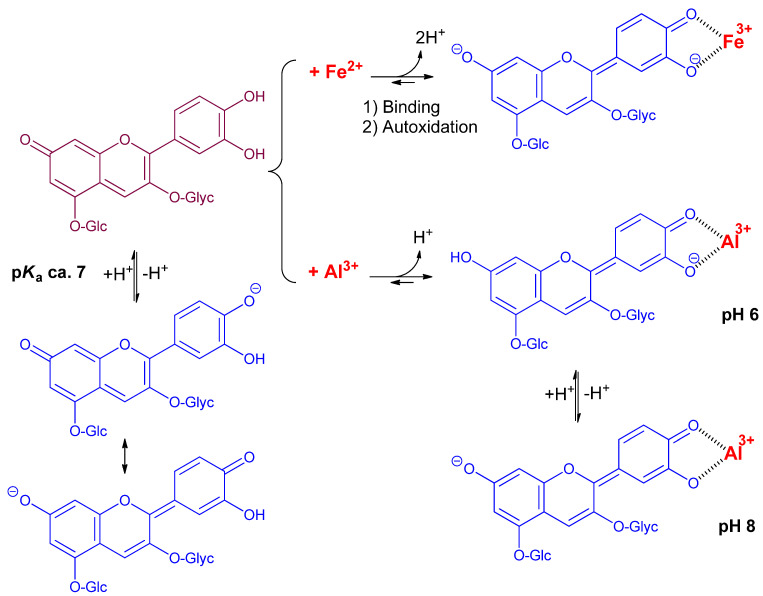
Metal–anthocyanin binding.

The pH dependence of the *λ*_max_ values in the pH range 6–8 for the free forms (Table S1) was consistent with the conversion of the neutral base (A_7_) to the anionic base (A_4′7_, proton loss from C4′-OH), in full agreement with the corresponding p*K*_a2_ value of the RC and PSP anthocyanins [7,13]. As Al^3+^ also triggers proton loss from C4′-OH, the bathochromic shift (BS) induced in the free forms by increasing the pH from 6 to 8 should be close to the one accompanying Al^3+^ binding at pH 6. Additionally, only small BS were expected upon Al^3+^ binding at pH 8. While the latter prediction was well-verified experimentally for all PSP pigments (Al^3+^-induced BS at pH 8 < 10 nm, Table S1), the former was not: indeed, the pH-induced BS (*ca*. 50 nm) were much larger than the Al^3+^-induced BS at pH 6 (from 16 nm for P4′ to 29 nm for P9b’). Moreover, bathochromism was clearly observed in the complexes’ visible band when the pH was increased from 6 to 8 (BS = 25–33 nm, Table S1). Hence, it can be proposed that C7-OH in the complexes was not dissociated at pH 6, and lost its proton when the pH is raised to 8 (Scheme 2). This proposal is consistent with the theoretical visible spectrum calculated on the Al(PB)_3_ complex, which, while involving 3 ligands undissociated at C7-OH, were found fully consistent with the experimental spectrum [8]. Moreover, comparing the neutral base of the 7-O-β-D-glucosyloxy-4′-hydroxyflavylium ion (proton loss from C4′-OH) with that of its 4′-O-β-D-glucosyloxy-7-hydroxyflavylium regioisomer (proton loss from C7-OH) also showed that the former displayed a *λ*_max_ value that was 20 nm higher [14] and a molar absorption coefficient almost 3 times as large. Finally, DFT calculations on pyranoanthocyanins [15] confirmed that the neutral base formed by proton loss from C4′-OH (A_4′_, a very minor species) displayed an intense absorption band at higher wavelengths than the major tautomer (proton loss from C7-OH). Overall, turning A_7_ into A_4′_ upon metal binding is actually expected to promote both bathochromism and hyperchromism.

Red cabbage anthocyanins with R_2_ = sinapoyl, whether mono- or diacylated, were distinct from the PSP anthocyanins and the other RC pigments in that the Al^3+^-induced BSs were larger, especially at pH 7 (36 and 52 nm for P5 and PB, respectively, vs. barely 5 and 8 nm for P2 and P4′, respectively) (Table 1, Figure 1 and Figure S1B). The *λ*_max_ values of the complexes were even higher than that of the anionic base. Thus, even if proton loss from C7-OH was not complete at pH 7 for the Al^3+^ complexes, the strong π-stacking interactions occurring between the cyanidin nucleus and R_2_ = sinapoyl and the concomitant torsion imposed between the B- and C-rings [8] effectively turned the color to an intense cyan hue (Figure 1) close to the one expressed by the major synthetic blue food colorants Brilliant Blue and indigotine.

**Figure 2:**
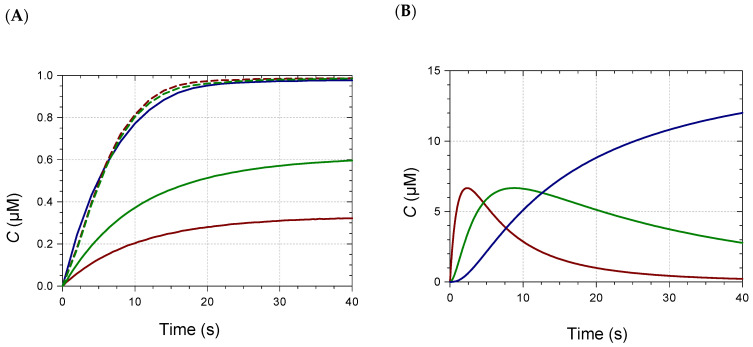

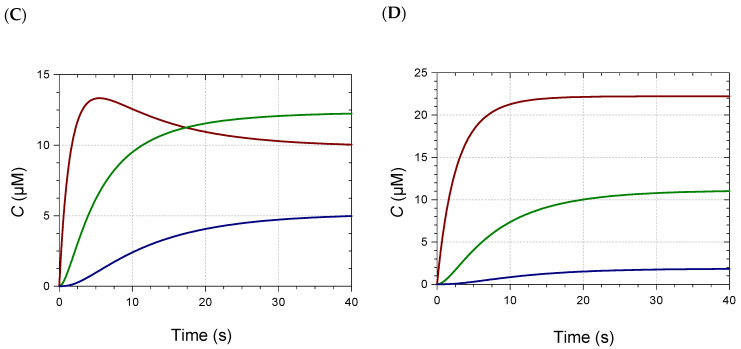
(**A**) Kinetics of Fe^2+^–Pigment B binding (pH 7, 25 °C). Pigment concentration = 50 µM, metal/pigment molar ratio = 0.05 (▬), 0.1 (▬), 0.3 (▬), 0.6 (▬ ▬), 1 (▬ ▬). (**B**–**D**) Speciation diagrams of Fe^2+^–Pigment B complexes. (**B**) 0.3 equiv. Fe^2+^. (**C**) 0.6 equiv. Fe^2+^. (**D**) 1 equiv. Fe^2+^. ML (▬), ML_2_ (▬), ML_3_ (▬). ML_n_: metal–ligand complex having a 1:n metal/ligand stoichiometry.

With Fe^2+^, the BSs were even larger: at pH 7, 87 and 76 nm for P5 and PB, respectively, vs. 25 nm for P2 (Table 1, Figure 1 and Figure S1B). As already reported for other Fe^2+^-polyphenol complexes [16,17], bound Fe^2+^ was rapidly autoxidized to Fe^3+^. This reaction was: (a) promoted by the higher affinity of catechols for Fe^3+^ (vs. Fe^2+^); (b) confirmed by the similarity of the final spectra, whether Fe^2+^ or Fe^3+^ was added [13]; and (c) consistent with the broad absorption band of the iron complexes and their high *λ*_max_, which both suggest ligand-to-Fe^3+^ charge transfer. However, iron autoxidation clearly followed metal binding. Indeed, despite the higher intrinsic affinity of catechols for Fe^3+^, anthocyanins bound Fe^2+^ much more rapidly in our model (Figure 2 and Figure S2) [13], as competition between anthocyanins and the phosphate anions for the metal was much less severe with Fe^2+^.

While the visible band of the Al^3+^ complexes of the RC anthocyanins showed the same pH dependence as for the PSP anthocyanins, the visible band of their Fe^3+^ complexes was remarkably insensitive to pH in the subgroup with R_2_ = sinapoyl (Figure S1B). In particular, the BS featuring iron–PB binding at pH 5.68 hit a record high of ca. 110 nm, i.e., twice as much as with Al^3+^ at the same pH. Hence, it can be proposed that C7-OH in the iron complexes is dissociated even at low pH (Scheme 2).

The colorimetric data of the PSP cyanidin glycosides and their Al^3+^ complexes (Table S2) provide additional evidence of the bluing effect induced by raising the pH from 6 to 8, or adding increasing Al^3+^ concentrations at a given pH. For comparison, a hue angle of 207.3 was recorded for the PB-Al^3+^ complex at pH 7, i.e., a close match for that of the synthetic triarylcarbonium colorant Brilliant Blue (h^0^ = 209.4), regarded as a reference for a vibrant cyan hue in the confectionary industry [8]. No such match was observed with the PSP anthocyanins and the best result recorded, i.e., the P6′-Al^3+^ complex at pH 8 (h^0^ = 221.7) remained off target. However, it confirmed that a single caffeoyl residue at R_1_ was sufficient to promote a strong bluing effect.

### 2.2. Kinetic Analysis and Stoichiometry of Metal Binding

When small volumes of concentrated Fe^2+^ or Al^3+^ aqueous solutions were added immediately after diluting the RC pigments into pH 6–8 phosphate buffers, a relatively fast metal–anthocyanin binding occurred (Figure 2 and Figure S2). Contrary to predictions based on steric hindrance, the observed trend was that acylation by noncoordinating HCA residues favored metal binding. For instance, nonacylated PA weakly bound Al^3+^ at pH 7 and 8 (weak spectral changes preventing the kinetic analysis), and strong Al^3+^–P2 binding only occurred at pH 8. By contrast, P5 strongly bound Al^3+^ at both pHs.

The minimal metal/ligand molar ratio to reach full binding (saturation of the visible band of the complex) is an indicator of the complex’s stoichiometry. This ratio lay between 1/3 and 2/3 for PA, PB, P2, and P5 (Figure 2 and Figure S3), as already observed with iron [9]. Thus, mixtures of 1:1, 1:2, and 1:3 complexes were expected in variable proportions according to the metal/ligand molar ratio. Consistently, in our recent work, the Al^3+^(PB)_3_ and Al^3+^(P6)_3_ complexes were evidenced by high-resolution mass spectrometry in a dilute ammonium acetate buffer, but not the Al^3+^(P3)_3_ homolog [8]. This was confirmed in the present work (Table S3). Under the same conditions, 1:1 and 1:2 iron–anthocyanin complexes were detected with P5 and PB (Table 2, Figure S4). With monoacylated P2, only the 1:1 complex was detected, whatever the M/L molar ratio between 1/6 and 1. The detection of 1:3 complexes is more challenging, as the corresponding ions must bear at least 3 charges for the *m/z* ratio to fall below 1500, the upper limit of detection. Satisfying agreements between experimental and theoretical *m/z* values were observed for the main ions and their major isotopes. Moreover, HRMS data were consistent with iron having a +3 oxidation degree in the complexes. For instance, the FeP5 monocation (exp. *m/z* 1208.2307, 1209.2352, 1210.2398) is proposed to be [P5 − 3H^+^ + Fe^3+^]^+^ (P5 referring to the flavylium cation, theoretical *m/z* 1208.2302, 1209.2335, 1210.2362). The corresponding complex involving Fe^2+^ would be [P5 − 2H^+^ + Fe^2+^]^+^ (*m/z* 1209.2381, 1210.2414, 1211.2440). Despite the possible match of the experimental spectrum with isotopes of the Fe^2+^ complex having one or two ^13^C-atoms, the intense signal at *m/z* 1208.2307 (Figure S4) clearly required an Fe^3+^ ion. Unexpectedly, varying the M/L molar ratio between 1/6 and 1 did not strongly impact the signal intensity of the 1:1 complex relative to free ligand (Table S4). Moreover, the signal intensities of the 1:1; 1:2, and 1:3 complexes could not be compared due to the charge-specific ion sensitivity of the MS detector.

A simple model assuming stepwise 1:1, 1:2, and 1:3 binding was tested to account for the kinetics of metal binding as a function of the metal concentration. To keep the number of adjustable parameters to a minimum, the rate constants of the first, second, and third steps were assumed to be 3*k* (3 available binding sites), 2*k* (2 available binding sites) and *k* (1 available binding site), respectively. Moreover, the molar absorption coefficients of the ML, ML_2_, and ML_3_ complexes (M = metal, L = ligand) were assumed to be *ε*, 2*ε,* and 3*ε*, respectively. Satisfactory curve-fittings of the *A*(670 nm) vs. time curves for different M/L molar ratios were thus obtained, leading to optimized values for parameters *k* and *ε*, and permitting the plotting of the time dependence of the concentrations of the 3 complexes (Table 3, Figure S5).

For given pigment and metal ion, the optimized *ε* values were fairly stable when the metal concentration was varied, which was satisfactory. This was less true for the optimized *k* values, which suggests that the model of independent binding steps was too simple. With the P5−Al^3+^ pair at pH 7, much better curve-fittings were actually obtained by implementing 3 optimizable rate constants in the model. Despite its crude approximations, the model offered a simple way to quantitatively assess the influence of pH, metal type, and acylation pattern on the rate of metal−anthocyanin binding. Overall, binding was faster at pH 8 than at pH 7, and faster with Fe^2+^ than with Al^3+^. Overall, under our conditions, acylation did not impede metal binding. Most importantly, pigments having R2 = sinapoyl (PB, P5) formed complexes with much higher molar absorption coefficients (typically, a factor 2–3), which was an obvious advantage for color development.

### 2.3. Competition between Metal Binding and Water Addition

Metal binding is accompanied by the removal of the B-ring’s phenolic protons. In other words, protons and metal ions compete for the O-atoms of the B-ring. Hence, lowering the pH gradually destabilizes the complexes and a minimal pH for the onset of metal binding is expected. Also, a weaker stability for the complexes could mean reversibility in their formation and thus competition with water addition to the free form (flavylium ion), which leads to color loss.

**Figure 3:**
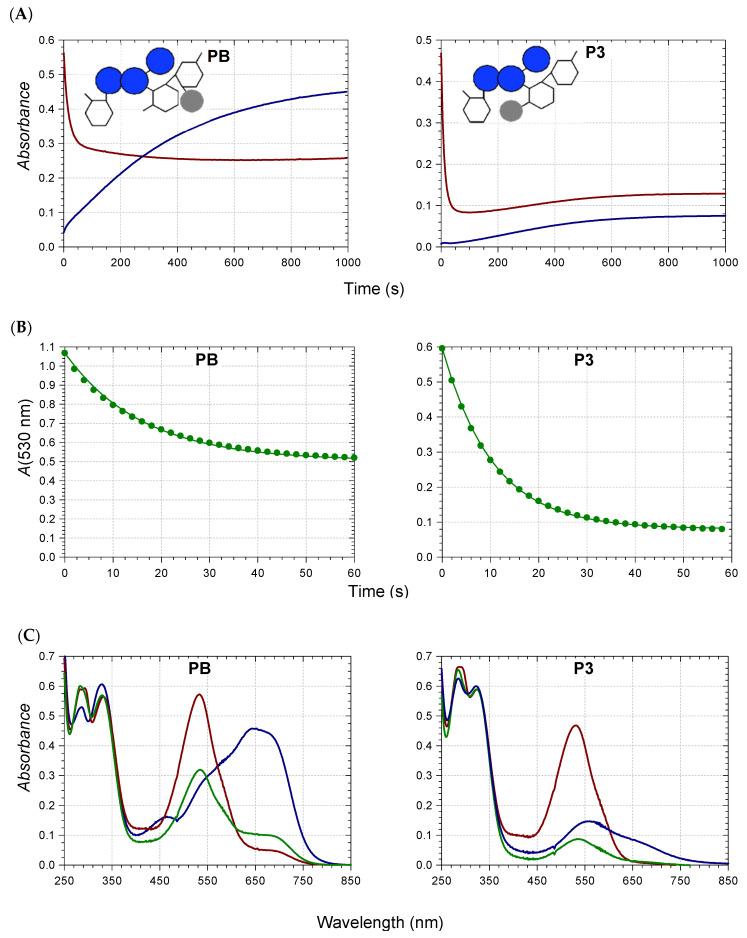

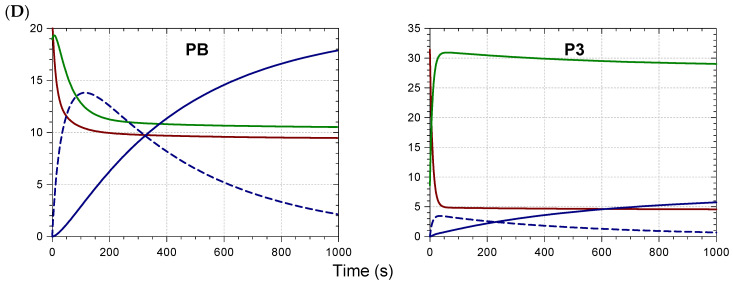
Iron–anthocyanin binding in a pH 4.24 acetate buffer (successive addition of pigment (40 µM) and Fe^2+^ (1 equiv.)), Left: Pigment B; right: Pigment 3. Simplified schemes are provided as a reminder of the key structural differences. (**A**) Spectral monitoring at 530 nm (free pigment, ▬) and at 670 nm (iron complex, ▬). (**B**) Control in the absence of iron (solid lines are the result of the curve-fitting procedure). (**C**) Spectra at time zero (▬), after ca. 1 min (▬) and at the end of the kinetic run (▬). (**D**) Time dependence of the species distribution: free colored forms (▬); free colorless forms (▬); Fe^2+^ complex (▬ ▬); Fe^3+^ complex (▬).

The influence of pH on iron binding was investigated with the two isomers PB and P3. For a given pH, the UV-VIS spectra were recorded immediately after pigment addition to buffer in the absence of Fe^2+^ and after maximal binding in the presence of Fe^2+^. A rise in visible absorbance in the range 600–700 nm, which is typical of metal binding, could be perceived with both pigments at pH ≥ 3 (data not shown). Although P3 and PB could not be clearly distinguished by the pH for the onset of iron binding, it was obvious that the hyperchromic and bathochromic shifts in mildly acidic solution (pH 4.24) were much more spectacular with PB than with P3 (Figure 3A,C). Part of the interpretation is rooted in the higher susceptibility of P3 to color loss by reversible water addition, which was both faster and more complete than for PB (Figure 3B). Indeed, when the pigment and Fe^2+^ (1 equiv.) were added to the acetate buffer, a sharp drop of visible absorbance at 530 nm (free pigment) was observed with P3 over the first minute, whereas the increase of visible absorbance at 670 nm (iron complex) was negligible (Figure 3A). In a second phase, the onset of iron binding occurred, and *A*(670 nm) slowly increased. In this case, the first step corresponding to the flavylium hydration (fast) was clearly decoupled from the second step (slow) of iron binding. By contrast, with PB, the drop of *A*(530 nm) over the first minute was limited and accompanied by a rise of *A*(670 nm), which was then amplified along the second phase. This is evidence that hydration and metal binding now compete from the beginning. It is interesting to note that if hydration was faster than metal binding in the case of P3, the higher affinity of the colored forms (vs. colorless forms) for the metal ion eventually permitted the reversal of the hydration equilibrium and the slow development of metal binding. However, even with PB, the intensity of the complex’s band in the pH range 3–6 remained much lower than in neutral solution, where it reached saturation even in the presence of substoichiometric iron concentrations. As the *λ*_max_ of the iron–PB complex’s visible band was pH-independent, the spectrum at pH 7 (Figure S1B) could be used to calculate the percentage of iron complex at equilibrium at pH 4.24: 47%. A similar calculation with P3 gave only 24%.

Attempts to fit the spectral changes observed at 530 nm (free form) and 670 nm (iron complex) to a simple kinetic model, assuming competition with water addition and reversible iron binding, failed. However, a more sophisticated scheme assuming reversible Fe^2+^ binding by the colored forms, followed by irreversible autoxidation of bound Fe^2+^ with concomitant formation of a Fe^3+^ complex (in equilibrium with the free species), provided perfect curve-fittings and acceptable rate constants for the different steps (Table S5). Overall, PB bound Fe^2+^ twice as rapidly as P3 did, and the Fe^2+^–PB complex seemed less susceptible to autoxidation than the Fe^2+^–P3 complex. Finally, the blue color development was much more intense with PB, as the molar absorption coefficient of the PB–Fe^3+^ complex in the blue domain was ca. twice as large than that of the P3–Fe^3+^ complex. These remarkable improvements only reflected the shift of the single sinapoyl residue from the 6 position of Glc-1 to the 2 position of Glc-2. This is a spectacular example of the crucial importance of strong anthocyanidin–hydroxycinnamoyl π-stacking interactions in the development of vibrant blue colors.

### 2.4. Long-Term Stability of the Metal Complexes

Acylation promoted a moderate increase in color stability upon heating at 50 °C, again with an advantage conferred on pigments with R2 = sinapoyl (Table S6, Figure S6). This trend was hugely emphasized after Fe^2+^ addition (0.6 equiv.), a clear indication that the sinapoyl residue on Glc-2 was very efficient for the long-term stabilization of the metal complexes. Again, the comparison between the P3 and PB isomers was striking: a 25% color loss of the Fe^2+^-P3 complex was reached in 15–20 min vs. 4.5 h for the Fe^2+^–PB complex. As already observed [9], the spectroscopic titration of the residual pigment (after acidification to pH 1 for total dissociation of the iron complex and total conversion of the colorless forms into the flavylium ion) provided a more contrasted picture: in the absence of added iron, acylation offered to protect, and this so-called “acylation paradox” [9] could be interpreted by assuming that the colorless forms (hemiketal and chalcones) were much less susceptible to oxidative degradation than the electron-rich anionic base. In other words, being more vulnerable to reversible water addition, nonacylated PA and anthocyanins having a single HCA residue at R1 (P1, P3) were protected against autoxidation at the cost of losing their color. However, after Fe^2+^ addition, autoxidation was strongly accelerated for PA because the corresponding iron complex was not stable enough, and the protection offered by iron binding was significant for P4 and PB only. In our recent work, adding Fe^2+^ also was shown to increase the yield in some major degradation products of PA and P1 [18]. In summary, strong π-stacking interactions within the complexes ensured an efficient iron sequestration, thus preventing the pro-oxidant activity of loosely bound iron ions [16]. The tight metal binding also explained the total inhibition of intramolecular acyl transfer, normally occurring when solutions of P4 or PB are heated [18].

## 3. Materials and Methods

Red cabbage and purple sweet potato anthocyanins were isolated by preparatory LC according to already-published procedures [1,2]. Their structures are presented in Scheme 1.

### 3.1. Metal-Binding Experiments

#### 3.1.1. Red Cabbage Anthocyanins

Metal–anthocyanin binding experiments were carried out according to previously reported procedures [13]. Fresh 5 mM solutions of Fe^2+^ and Al^3+^ were respectively prepared from FeSO_4_–7H_2_O and AlCl_3_–6H_2_O (Sigma-Aldrich, St-Quentin Fallavier, France) in 1 mM aqueous HCl. Concentrated stock solutions of pigment (5 mM) were prepared in 50 mM aqueous HCl. Absorption spectra were recorded on an Agilent 8453 diode-array spectrometer in thermostated and magnetically stirred quartz cuvettes (pathlength = 1 cm). The following solutions were directly added to the cuvette in this order: 2 mL of 10 mM phosphate buffer (pH 7 or 8), 20 μL of anthocyanin stock solution and, after a few seconds (negligible formation of colorless forms), a small volume of the 5 mM Fe^2+^ or Al^3+^ solution (final iron/anthocyanin molar ratio = 1 or 2). The full UV-VIS spectra were recorded in kinetic mode for 1 to 2 min. For an optimal sensitivity, the detection in the visible range was set at 550 or 610 nm for Al^3+^ (close to the complex’s *λ*_max_) and at 670 nm for Fe^2+^ (charge-transfer contribution of the Fe^3+^ complexes). The hyperchromic and bathochromic shifts were calculated from the initial (free ligand) and final (metal complex) spectra as (*A*_max,f_ − *A*_max,0_)/*A*_max,0_ and *λ*_max,f_ − *λ*_max,0_, respectively.

Binding experiments were also carried out in the pH range 2–6 (50 mM acetate buffer) to determine the pH for the onset of metal binding and investigate the competition with water addition to the flavylium ion.

#### 3.1.2. Purple Sweet Potato Anthocyanins

The anthocyanin isolates were diluted to a 50 µM concentration in buffers of pH 6 (0.1 M sodium acetate), 7, and 8 (0.25 M TRIS). Small volumes of concentrated Al_2_(SO_4_)_3_ solutions were then added to reach metal/anthocyanin ratios of 0.5, 1, and 5. Samples were equilibrated for 30 min at room temperature in the dark prior to analysis (in triplicates). UV-VIS spectra were collected from 380 to 700 nm using 300 µL samples in poly-D-lysine-coated polystyrene 96-well plates with a SpectraMax 190 Microplate Reader (Molecular Devices, Sunnyvale, CA, USA).

### 3.2. Kinetic Analyses

The kinetic curves were analyzed with the Scientist software (Micromath, St. Louis, MO, USA). Sets of differential equations characteristic of the different kinetic processes (metal binding, water addition to the flavylium ion) were implemented in the models, as well as initial concentrations. Optimized values for the adjustable parameters (rate constants, molar absorption coefficients) and their standard deviations are reported.

### 3.3. Colorimetric Data

Color characteristics were expressed in the L*a*b* coordinates. L* corresponds to the light intensity, varying from 0 (no light) to 100. Parameters a* and b* quantify the contribution of four colors: green (−a*), red (+a*), blue (−b*) and yellow (+b*). With RC anthocyanins, the method to generate the L*a*b* coordinates and the corresponding color patches was as described in our recent work [13]. With PSP anthocyanins, the colorimetric data were generated as previously reported [2].

### 3.4. High-Resolution Mass Spectrometry (HRMS)

Stock solutions of anthocyanins P2, PB, and P5 were diluted to 0.1 mM in a 50 mM ammonium acetate buffer at pH 7. A 0.5 µL volume was injected in the same solvent (flow injection analysis) over 1 min into an Orbitrap Exploris 480 mass spectrometer Exploris 480 (Thermo Fisher Scientific, Waltham, MA, USA) equipped with an H-ESI source. A static spray voltage of –3.5 kV in positive mode and of 2.5 kV in negative mode was applied with an ion-transfer tube temperature of 280 °C, a vaporizer temperature of 300 °C, and a N_2_ sheath gas pressure of 40 psi. The full scan was recorded at a resolution of 24 × 10^4^ and corrected with an internal mass calibrant. Ions were searched between *m/z* 400 and 1600. The elution lasted ca. 0.3 min and an average spectrum was determined with the Freestyle 1.6 software based on the 20–30 spectra of intensities higher than 10%. A targeted detection of the Fe^2+^, Fe^3+^, and Al^3+^ complexes of stoichiometries 1:1, 1:2, and 1:3 and with charges ranging from −3 to +4 was carried out. Based on proposed raw formulae, the theoretical isotopic patterns were calculated with the https://www.chemcalc.org/ web tool (accessed on 2 March 2021).

### 3.5. Thermal Degradation

Thermal degradation was performed at pH 7 and 50 °C in a thermostated water bath. The pigments were diluted to 50 μM in the phosphate buffer at 50 °C and UV-VIS spectra were recorded over 24 h. The residual fraction of color species at pH 7 (a mixture of neutral and anionic bases) was determined at *λ*_max_ as % Color = 100 × *A*_λmax_(*t*)/*A*_λmax_(*t* = 0). Aliquots of 1.5 mL were taken up at time zero, at regular time intervals over 8 h, and finally at *t* = 24 h. They were cooled down, acidified to pH 1, and stabilized at room temperature for 15 h (nonacylated anthocyanins) to 48 h (diacylated anthocyanins) (to ensure complete regeneration of the flavylium ion from the colorless forms). The absorption spectra were then recorded and the residual fraction of flavylium ion was calculated as % AH^+^ = 100 × *A*_λmax_(*t*)/*A*_λmax_(*t* = 0) and plotted as a function of time. The time periods for a 25% color loss, or a 25% pigment loss (noted *t*_25_), were determined from the theoretical curves generated by exponential curve-fitting.

## 4. Conclusions

Anthocyanins acylated by hydroxycinnamic acid residues are prone to develop inter- and intramolecular π-stacking interactions that make the flavylium nucleus less vulnerable to water addition. However, the acylation site plays a critical role, and among the red cabbage anthocyanins, it is clear that the sinapoyl residue at C2-OH of Glc-2 is by itself sufficient to provide resistance to water addition. Enhanced π-stacking interactions result from the ideal positioning of the sinapoyl residue (external Glc + C2 position), which probably reaches an optimal compromise between rigidity and flexibility.

The present work shows that optimal cyaniding–hydroxycinnamoyl π-stacking interactions can also exert a critical and favorable influence on metal binding. Indeed, only the metal complexes having R_2_ = sinapoyl (pigments B, 4, 5, and 6) are deeply blue and thermally stable over long periods of time. Not only the sinapoyl residue (although noncoordinating by itself) does not compromise metal binding by steric hindrance, it also shifts the visible band to the blue domain and increases its intensity, and stabilizes the metal complexes and prevents the release of free pro-oxidant iron in the medium.

Given the diversity of metal ions possibly binding anthocyanins in plants, other metal complexes of acylated cyanidin glycosides might also be tested for their coloring properties. In summary, anthocyanins having a 3′,4′-dihydroxysubstitution (cyanidin, delphinidin and petunidin glycosides) with adequately located hydroxycinnamoyl residues offer bright prospects for further development as natural food colorants.

## Figures and Tables

**Table 1:** Spectral characteristics of the metal complexes of red cabbage and purple sweet potato anthocyanins (1 equiv. metal ion). Δ*λ*_max_ = *λ*_max_(+metal) − *λ*_max_(no metal). Δ*A* = *A*(+metal) − *A*(no metal).

		Fe^2+^				Al^3+^			
Red Cabbage Pigments		**PA**	**P2**	**P5**	**PB**	**PA**	**P2**	**P5**	**PB**
pH 6	*λ*_max_(+metal) (nm)	559	569	640	656	no binding	571	587	629
	Δ*λ*_max_ (nm)	21	25	87	76		27	34	49
	Δ*A*/*A*(no metal) at *λ*_max_ (%)	−25	−15	3	65		−15	−9	16
pH 7	*λ*_max_(+metal) (nm)	596	592	646	656	583	585	625	637
	Δ*λ*_max_ (nm)	14	−4	55	71	4	5	34	52
	Δ*A*/*A*(no metal) at *λ*_max_ (%)	−7	−3	19	43	3	−1	17	25
Purple Sweet Potato Pigments						**P4′**	**P6′**	**P7′**	**P9b′**
pH 6	*λ*_max_(+metal) (nm)	/	/	/	/	563	574	575	581
	Δ*λ*_max_ (nm)	/	/	/	/	16	26	23	29
pH 7	*λ*_max_(+metal) (nm)	/	/	/	/	580	591	598	598
	Δ*λ*_max_ (nm)	/	/	/	/	8	21	22	16

**Table 2:** Ions detected for the iron complexes of pigments P5 = Cya-3-(Fl)Glc-2-(Sp)Glc-3-Glc and PB = Cya-3-Glc-2-(Sp)Glc-3-Glc from red cabbage (metal/ligand molar ratio = 1).

Compound	Formula	Th. *m/z* (rel. abundance) ^(^^a)^	Exp. *m/z* (rel. abundance) ^(b^^)^	*δ* (ppm)
P5^+^	C_54_H_59_O_28_	1155.3187	1155.3198	1.0
[P5 − 3H^+^ + Fe^3+^]^+^	C_54_H_56_O_28_Fe	1208.2302 (100) 1209.2335 (62.4) 1210.2362 (25.2)	1208.2307 (100) 1209.2352 (98) 1210.2397 (46)	0.4 1.4 2.9
[P5 − 2H^+^ + Fe^3+^]^2+^	C_54_H_57_O_28_Fe	604.6188 (100) 605.1204 (62.0) 605.6217 (24.9)	604.6189 (100) 605.1211 (83) 605.6227 (32)	0.2 1.2 1.7
[2P5 − 2H^+^ + Fe^3+^]^3+^	C_108_H_116_O_56_Fe	788.1856 (84.1) 788.5199 (100) 788.8542 (69.8)	788.1836 (87) 788.5183 (100) 788.8537 (35)	2.5 2.0 0.6
PB^+^	C_44_H_51_O_25_	979.2714	979.2722	0.8
[PB − 3H^+^ + Fe^3+^]^+^	C_44_H_48_O_25_Fe	1032.1829 (100) 1033.1862 (51.4) 1034.1887 (18.4)	1032.1836 (100) 1033.1888 (71) 1034.1921 (25)	0.7 2.5 3.3
[PB − 2H^+^ + Fe^3+^]^2+^	C_44_H_49_O_25_Fe	516.5951 (100) 517.0967 (51.1) 517.5980 (18.2)	516.5952 (100) 517.0969 (53) 517.5982 (16)	0.2 0.4 0.4
[2PB − 3H^+^ + Fe^3+^]^2+^	C_88_H_99_O_50_Fe	1005.7273 (100) 1006.2289 (98.5) 1006.7303 (59.0) 1007.2317 (26.2)	1005.7277 (63) 1006.2304 (100) 1006.7320 (80) 1007.2343 (45)	0.4 1.5 1.7 2.6
[PB − 2H^+^]^−^	C_44_H_49_O_25_	977.2563	977.2570	0.7
[PB − 5H^+^ + Fe^3+^]^−^	C_44_H_46_O_25_Fe	1030.1683 (100) 1031.1716 (51.4) 1032.1741 (18.4)	1030.1684 (100) 1031.1723 (53) 1032.1752 (16)	0.1 0.7 1.1
[PB − 6H^+^ + Fe^3+^]^2−^	C_44_H_45_O_25_Fe	514.5806 (100) 515.0822 (51.1) 515.5834 (18.2)	514.5807 (100) 515.0822 (47) 515.5837 (12)	0.2 0.0 0.6
[2PB − 7H^+^ + Fe^3+^]^2−^	C_88_H_95_O_50_Fe	1003.7128 (100) 1004.2143 (98.5) 1004.7157 (58.9) 1005.2171 (26.2)	1003.7124 (100) 1004.2153 (93) 1004.7166 (65) 1005.2180 (40)	0.4 1.0 0.9 0.9

^(a)^ Relative abundances calculated on the website https://www.chemcalc.org (accessed on 2 March 2021). ^(b)^ Relative abundances estimated with the Freestyle software (Thermo Fisher Scientific, Waltham, MA, USA).

**Table 3:** Kinetic analyses of metal–anthocyanin binding according to the stepwise formation of ML, ML_2_, and ML_3_ complexes (25 °C, monitoring at 670 nm). ML_n_: metal–ligand complex having a 1:n metal/ligand stoichiometry. For simplicity, the successive binding steps are described by rate constants 3*k*, 2*k,* and *k*, respectively, and each bound ligand by the same molar absorption coefficient *ε*. Some repetitions are shown. Values between brackets are standard deviations for the curve-fitting procedure.

M/Pigment	*k* (×10^3^) (M^−1^ s^−1^)	*ε* (×10^3^) (M^−1^ cm^−1^)
Pigment A + Fe^2+^, pH 7		
0.3	4.1 (0.1)	9.5 (0.1)
0.6	4.0 (0.3), 3.2 (0.2), 3.7 (0.2)	8.6 (0.2), 8.8 (0.2), 9.0 (0.1)
1.0	3.1 (0.3), 4.0 (0.4), 3.7 (0.3)	9.9 (0.4), 8.4 (0.2), 9.1 (0.2)
Pigment B + Fe^2+^, pH 7		
0.3	2.03 (0.15), 4.16 (0.36)	38.3 (1.4), 26.1 (0.4)
0.6	6.17 (0.15), 2.32 (0.13)	22.6 (0.1), 21.7 (0.2)
1.0	0.90 (0.02), 1.18 (0.04), 1.03 (0.04)	23.6 (0.1), 23.1 (0.1), 23.2 (0.1)
Pigment A + Al^3+^, pH 7 *Weak binding*		
Pigment B + Al^3+^, pH 7		
0.3	0.81 (0.01)	15.4 (0.1)
0.6	0.92 (0.01), 0.47 (0.01)	15.0 (0.1), 15.6 (0.1)
1	0.42 (0.01), 0.51 (0.01), 0.39 (0.01)	14.5 (0.1), 16.0 (0.1), 14.6 (0.1)
P5 + Fe^2+^, pH 7		
0.3	2.7 (0.1)	24.5 (0.1)
0.6	2.5 (0.1)	22.1 (0.1)
1	3.1 (0.1)	24.9 (0.1)
P5 + Fe^2+^, pH 8		
0.3	13.6 (0.3)	13.5 (0.1)
0.6	6.4 (0.1)	12.9 (0.1)
1	6.6 (0.3)	12.8 (0.1)
P2 + Fe^2+^, pH 7		
0.3	16.6 (0.8)	6.7 (0.1)
0.6	4.4 (0.1)	8.7 (0.1)
1	2.5 (0.1)	10.0 (0.1)
P2 + Fe^2+^, pH 8		
0.3	14.1 (1.5)	12.4 (0.4)
0.6	9.1 (0.7)	14.0 (0.3)
1	5.5 (0.4)	13.6 (0.2)
P5 + Al^3+^, pH 7 (a)		
0.3	3.7 (0.1), 1.3 (0.1), 0.39 (0.05)	16.7 (0.2)
0.6	0.58 (0.03), 0.14 (0.06) (b)	17.6 (1.1)
1	0.50 (0.01) (c)	14.0 (0.1)
P5 + Al^3+^, pH 8		
0.3	5.5 (0.1)	10.3 (0.1)
0.5	6.1 (0.1)	8.9 (0.1)
1	1.7 (0.1)	7.4 (0.1)
P2 + Al^3+^, pH 7 *Weak binding*		
P2 + Al^3+^, pH 8		
0.3	4.1 (0.1)	3.4 (0.1)
0.6	3.1 (0.1)	3.7 (0.1)
1	1.2 (0.1)	3.8 (0.1)

Note: (a) 3 optimizable rate constants; (b) *k*_3_ = 0; (c) *k*_2_ = *k*_3_ = 0.

## Data Availability

The data that support the findings of this study are available from the corresponding author upon reasonable request.

## Supplementary Materials

Supplementary materials can be found at https://www.mdpi.com/article/10.3390/ijms22094551/s1.
