# Supplementary material for: Acylated Anthocyanins from Red Cabbage and Purple Sweet Potato Can Bind Metal Ions and Produce Stable Blue Colors
Source: Int J Mol Sci. 2021 Apr 27;22(9):4551. doi: 10.3390/ijms22094551 (PMC8123718; doi:10.3390/ijms22094551)
Supplement: Supplementary file 1 [file ijms-22-04551-s001.zip › ijms-1162477-SI.pdf]

Acylated anthocyanins from red cabbage and purple sweet potato can bind metal ions and produce stable blue colors

Julie-Anne Fenger,[a]* Gregory T. Sigurdson,[b] Rebecca J. Robbins,[c] Thomas M. Collins,[d] M. Mónica Giusti,[b] Olivier Dangles[a]*

Supplementary Information

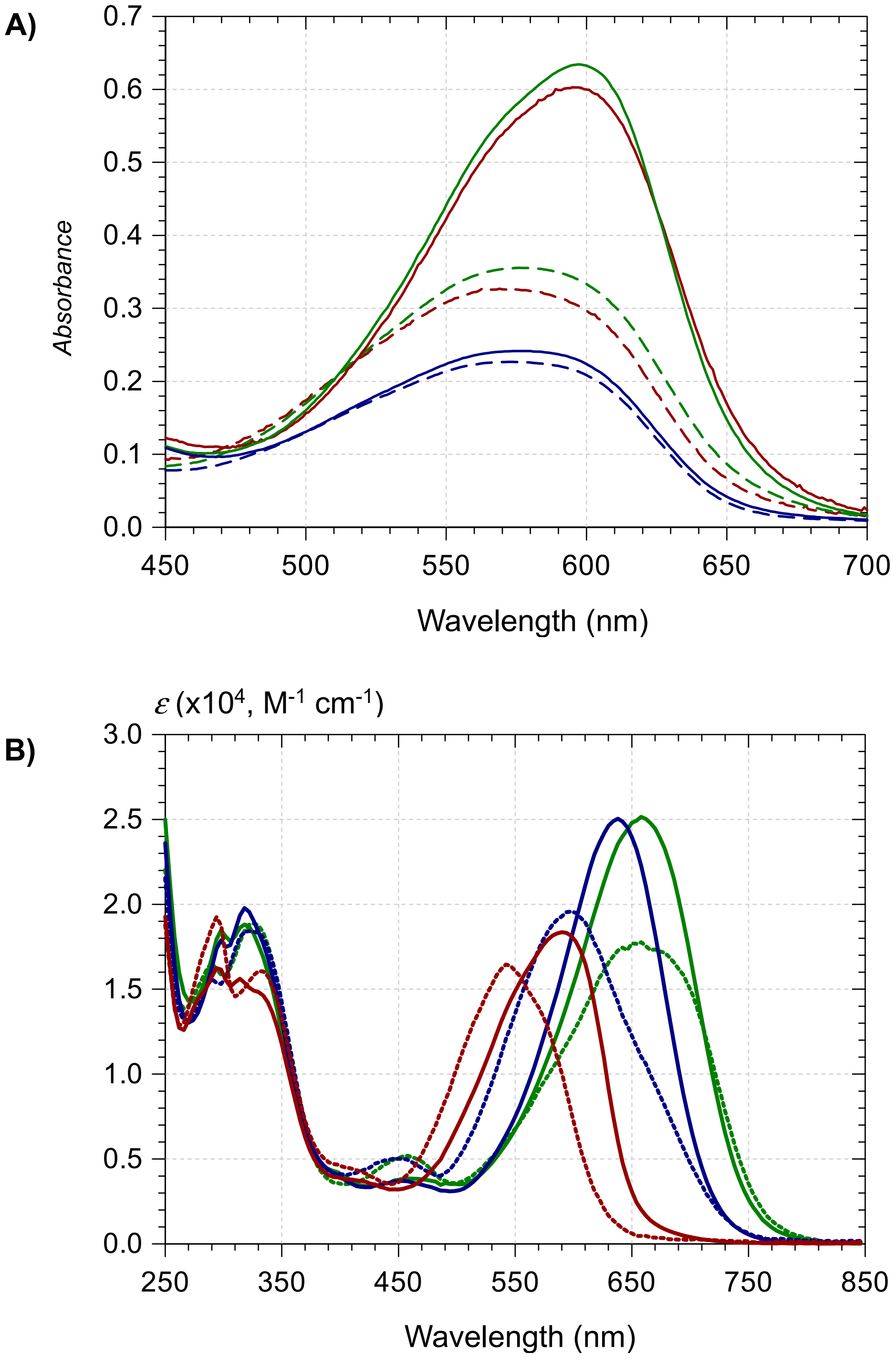

Fig. 1-SI. A) Visible spectra of PSP anthocyanins (solid lines) and their Al^{3+} complexes (dotted lines, 1 equiv. Al^{3+}) recorded at pH 7 30 min after sample preparation. Pigment 4' (▬, acyl = Fl), Pigment 6' (▬, acyl = Cf), Pigment 7 (▬, acyl = Cf, Cf). **B)** UV-visible spectra of pigment B at pH 7.0 (solid curves) and at pH 5.68 (dot curves). red: control, green: + Fe^{2+} (1 equiv.), blue: + Al^{3+} (1 equiv.).

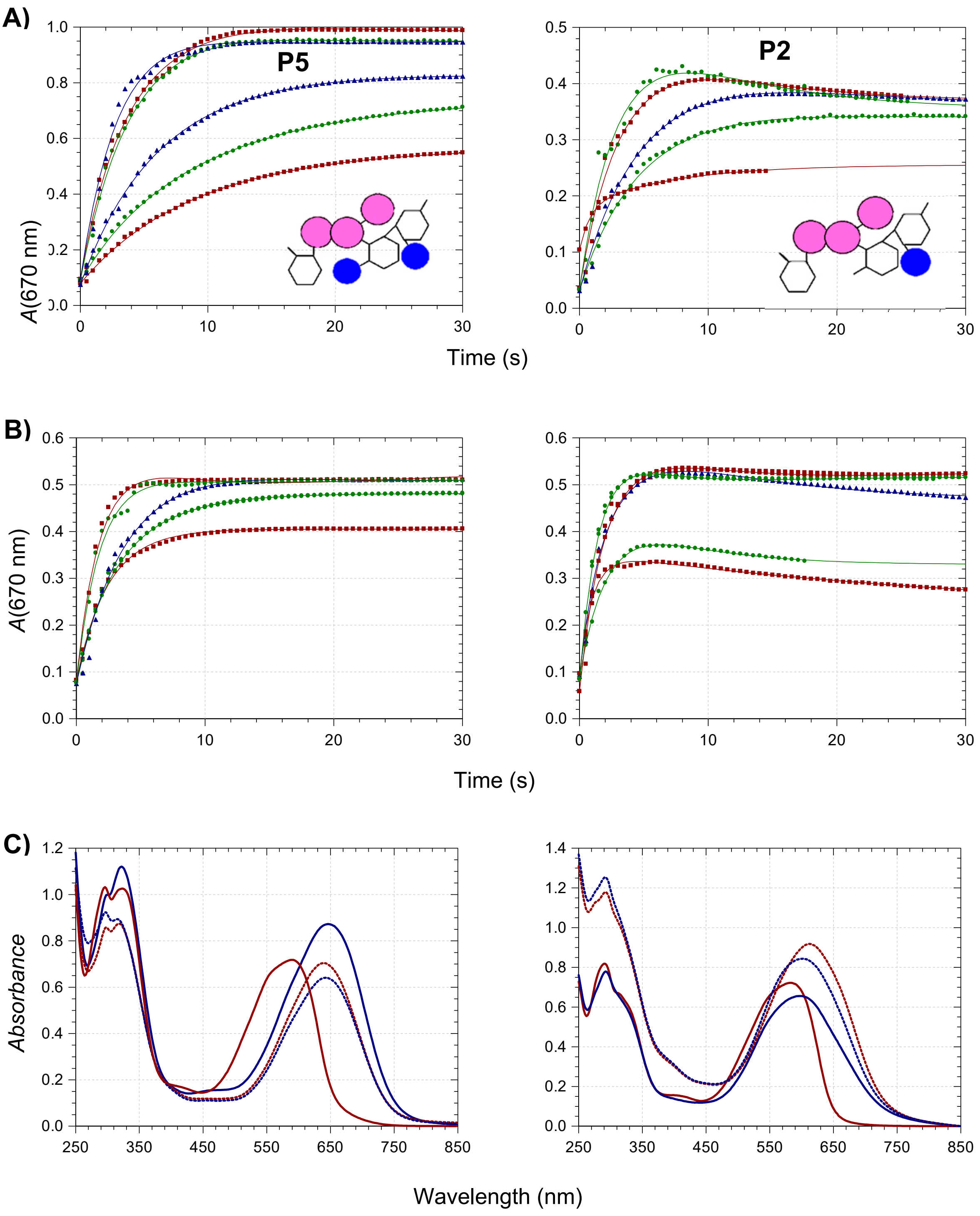

Fig. 2-SI. Iron - anthocyanin binding (successive addition of pigment (40 µM at pH 7, 20 µM at pH 8) and Fe^{2+}). **A & B)** Kinetic monitoring at pH 7 (A) or pH 8 (B). Iron/pigment molar ratio (from bottom to top curves) = 0.1, 0.3, 0.6, 1, 1.5, 2 (Pigment 5, pH 7), 0.3, 0.6, 1, 2, 4 (Pigment 2, pH 7), 0.1, 0.3, 0.6, 1, 2 (Pigments 2 and 5, pH 8). **C)** Spectra at time zero (▬) and at the end of the kinetic run (▬). 1 equiv. Fe^{2+}. Solid curves: pH 7. Dashed curves: pH 8.

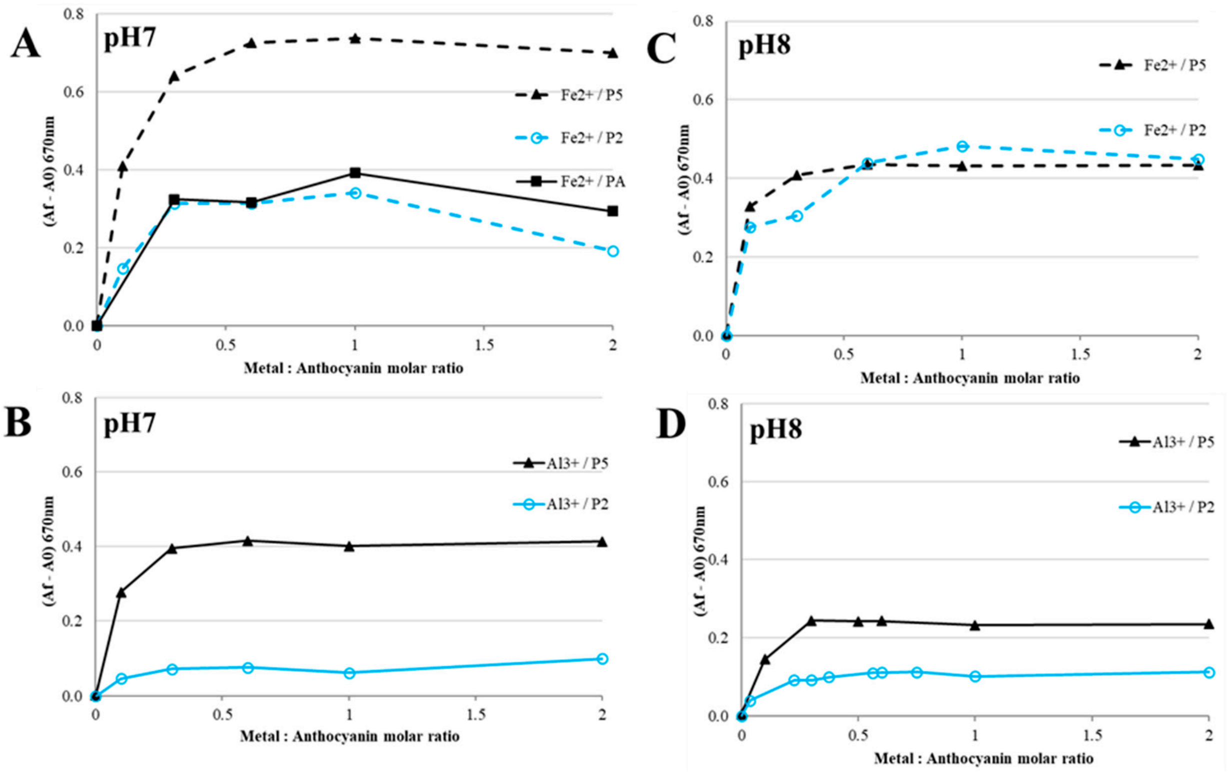

Fig. 3-SI. Amplitude of the complex's visible band as a function of the metal/ligand molar ratio. Pigment 2 (○), Pigment 5 (▲).

A)

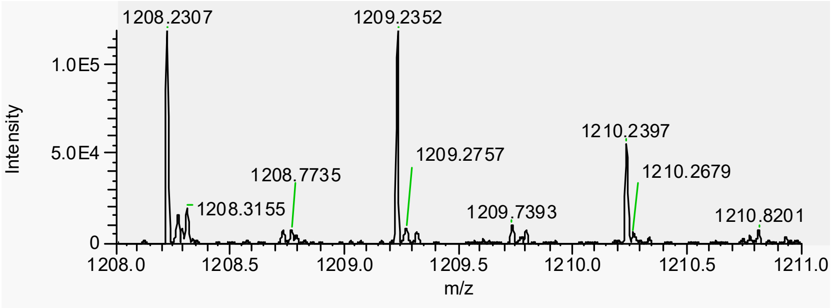

$[P5 - 3H^{+} + Fe^{3+}]^{+}$

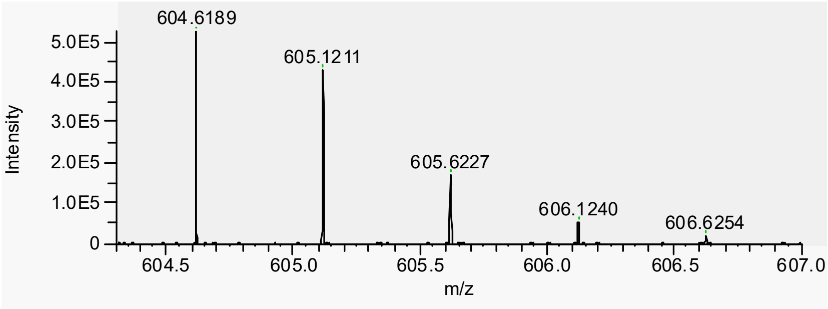

$[P5 - 2H^{+} + Fe^{3+}]^{2+}$

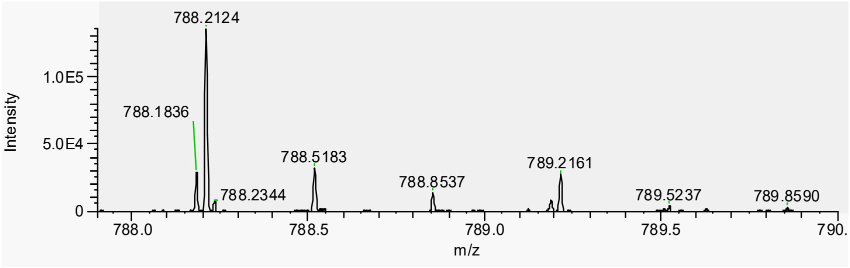

$[2P5 - 2H^{+} + Fe^{3+}]^{3+}$

(+ unidentified signals at *m/z* 788.2124 and 789.2161)

B)

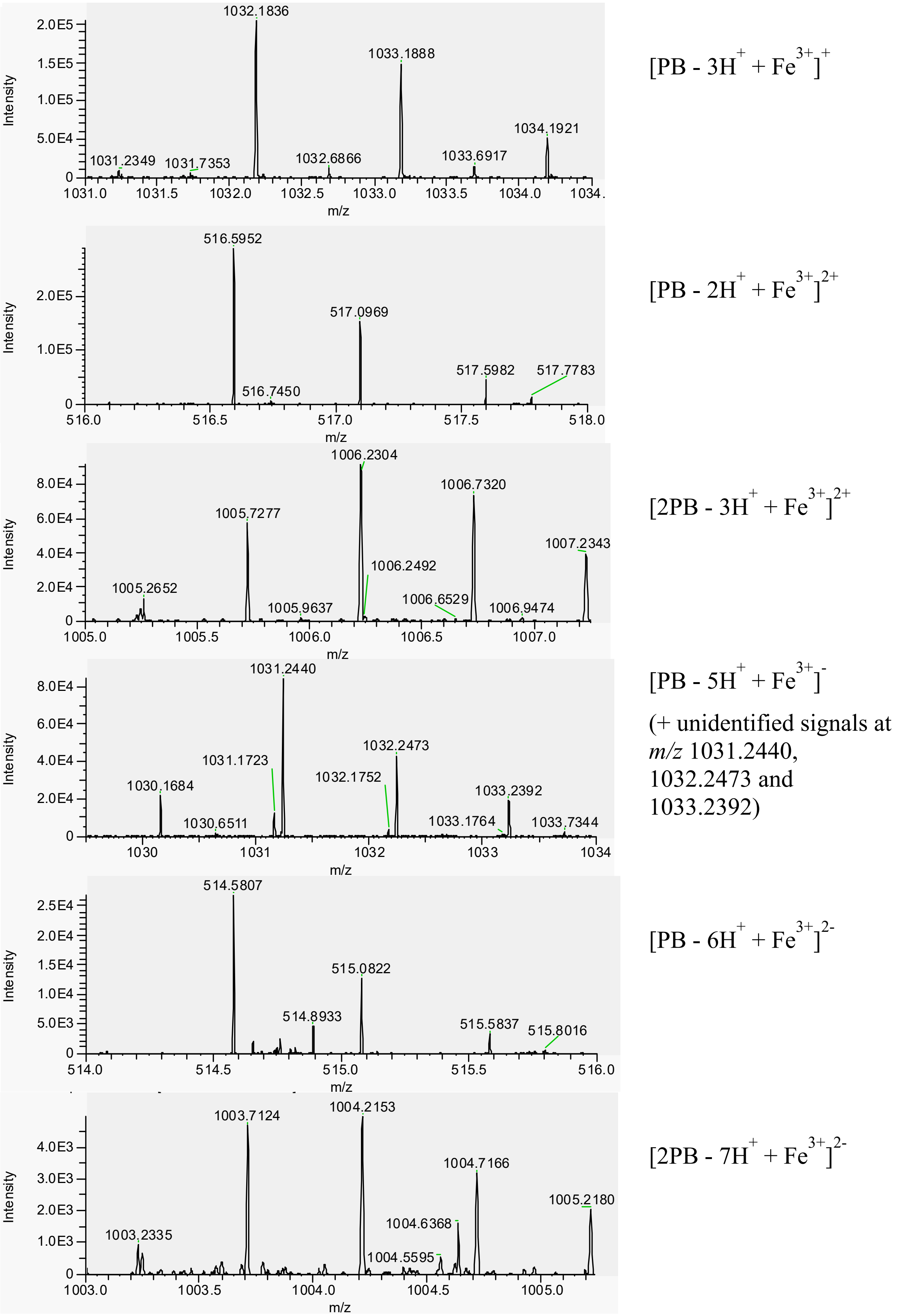

Fig. 4-SI. HRMS analyses of iron – anthocyanin complexes (pH 7 ammonium acetate buffer, metal/ligand molar ratio = 1). **A)** Pigment 5, **B)** Pigment B.

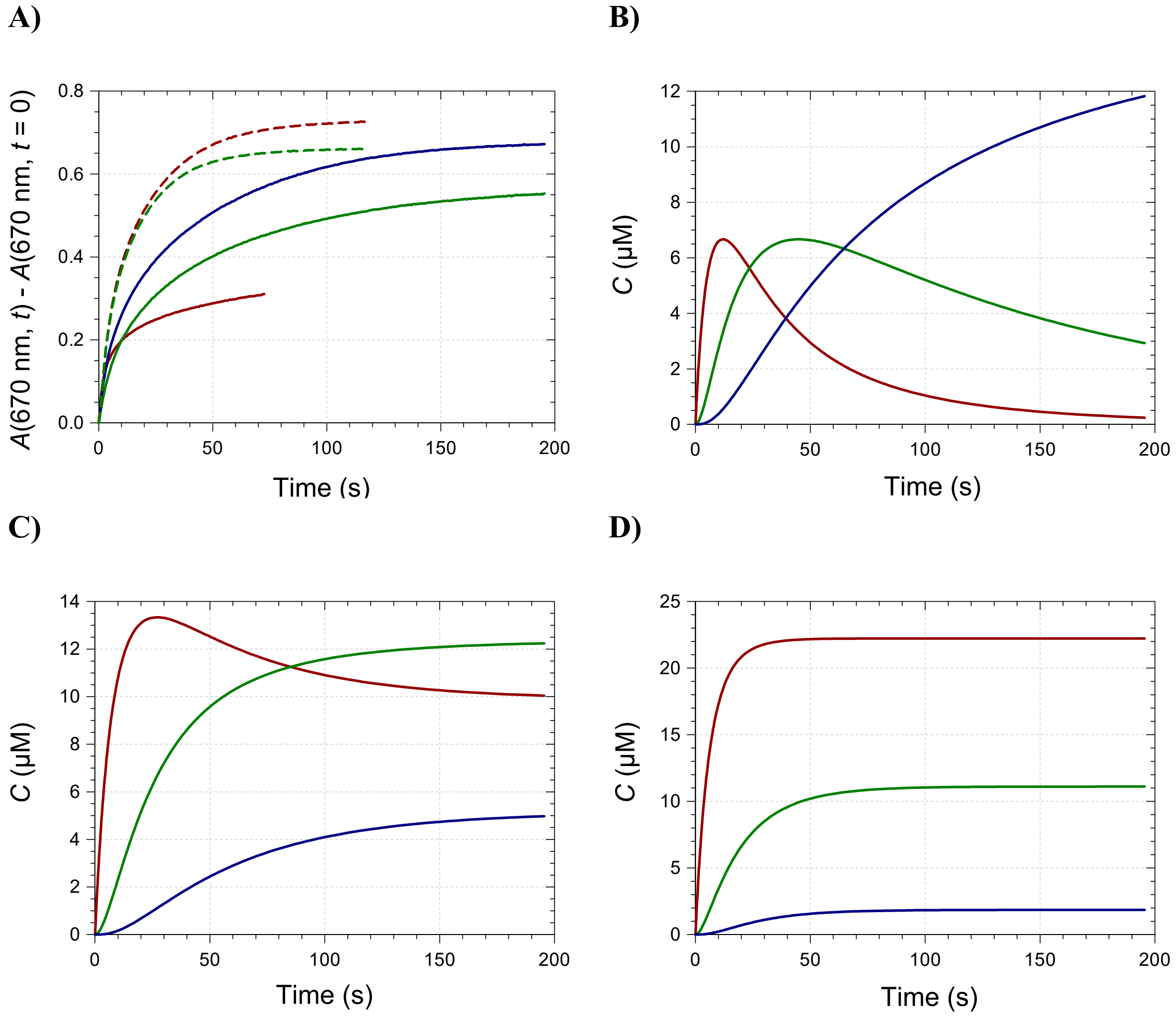

Fig. 5-SI. A) Kinetics of Al^{3+} – pigment B binding (pH 7, 25°C). Pigment concentration = 50 µM, metal / pigment molar ratio = 0.1 (▬), 0.3 (▬), 0.6 (▬), 1 (▬ ▬), 2 (▬ ▬). **B-D)** Speciation diagrams of Al^{3+} - pigment B complexes. **B)** 0.3 equiv. Al^{3+}. **C)** 0.6 equiv. Al^{3+}. **D)** 1 equiv. Al^{3+}. ML (▬), ML_2 (▬), ML_3 (▬). ML_n: metal-ligand complex having a 1:n metal/ligand stoichiometry.

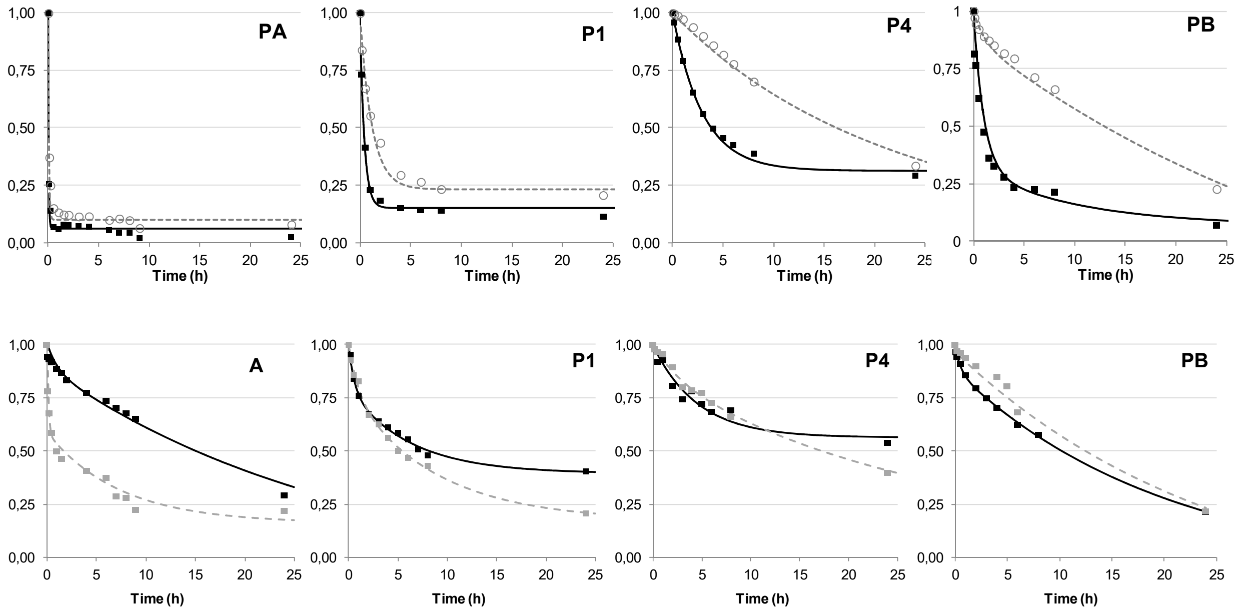

Fig. 6-SI. Rate of color loss (*top*) and pigment loss (*bottom*) in solutions of Pigments A, 1, 4 and B at pH 7, 50°C, in the absence (black plain line) or presence (grey dashed line) of 0.6 equiv. Fe^{2+}.

Table 1-SI. Wavelengths of maximal visible absorption (λ_{max}, in nm) of PSP anthocyanins (50 μM) in the absence or presence of Al^{3+}, 25°C, n = 3.

Pigment	No Al^{3+}	0.5 equiv. Al^{3+}	1 equiv. Al^{3+}	5 equiv. Al^{3+}
pH 6				
P4’	547 (2)	562 (1)	563 (1)	569 (1)
P6’	548 (3)	572 (2)	574 (1)	581 (1)
P7’	552 (1)	570 (0)	575 (3)	584 (1)
P9b’	552 (3)	584 (1)	581 (1)	589 (7)
pH 7				
P4’	572 (2)	583 (1)	580 (0)	576 (3)
P6’	570 (1)	591 (3)	591 (2)	590 (1)
P7’	576 (1)	594 (0)	598 (1)	595 (0)
P9b’	582 (3)	598 (2)	598 (1)	597 (5)
pH 8				
P4’	598 (0)	596 (1)	596 (1)	594 (4)
P6’	600 (1)	602 (2)	601 (1)	597 (3)
P7’	603 (0)	603 (0)	601 (0)	602 (1)
P9b’	604 (1)	607 (1)	606 (1)	606 (2)

Table 2-SI. Hue angle (h^0) of PSP anthocyanins (50 μM) treated with Al^{3+}, 25°C, *n* = 3.

Pigment	No Al^{3+}	0.5 equiv. Al^{3+}	1 equiv. Al^{3+}	5 equiv. Al^{3+}
pH 6				
P4'	332.1 (1.1)	308.7 (1.2)	302.6 (0.9)	295.7 (0.9)
P6'	325.1 (0.6)	277.7 (1.5)	273.4 (0.4)	269.1 (0.2)
P7'	311.6 (0.2)	285.9 (0.2)	280.7 (2.2)	271.4 (0.0)
P9b'	308.1 (0.8)	265.3 (0.2)	265.1 (0.2)	263.3 (0.5)
pH 7				
P4'	291.6 (0.2)	271.6 (0.4)	272.3 (0.6)	269.4 (1.7)
P6'	293.0 (0.2)	253.8 (0.4)	247.8 (0.2)	249.9 (0.3)
P7'	285.9 (0.1)	261.2 (1.1)	254.6 (0.4)	249.6 (0.4)
P9b'	284.0 (0.3)	247.7 (1.2)	248.2 (0.9)	248.7 (1.2)
pH 8				
P4'	241.6 (0.2)	238.4 (0.3)	239.3 (0.2)	239.7 (0.6)
P6'	245.5 (0.2)	221.7 (0.1)	228.0 (0.7)	231.8 (0.3)
P7'	245.1 (0.1)	235.8 (0.3)	239.2 (1.2)	238.5 (0.5)
P9b'	243.4 (0.4)	232.9 (0.2)	234.0 (0.3)	235.4 (0.3)

a) h^0(PB-Al^{3+} complex, pH 7) = 207.3 *vs*. 209.4 for Brilliant Blue [8]

Table 3-SI. Ions detected for the aluminum complexes of Pigment B (metal/ligand molar ratio = 1).

Compound	Formula	Th. *m/z* [a])	Exp. *m/z*	*δ* (ppm)
PB^{+}	$C_{44}H_{51}O_{25}$	979.2714	979.2722	0.8
$[PB + Al^{3+} - 3H]^{+}$	$C_{44}H_{48}O_{25}Al$	1003.2295	1003.2301	0.6
$[PB + Al^{3+} - 2H^{+}]^{2+}$	$C_{44}H_{49}O_{25}Al$	502.1184	502.1184	0.0
$[2PB + Al^{3+} - 3H^{+}]^{2+}$	$C_{88}H_{99}O_{50}Al$	991.2504	991.2510	0.6
$[PB - 2H^{+}]^{-}$	$C_{44}H_{49}O_{25}$	977.2563	977.2570	0.7
$[PB + Al^{3+} - 5H^{+}]^{-}$	$C_{44}H_{46}O_{25}Al$	1001.2149	1001.2151	0.2
$[PB + Al^{3+} - 6H^{+}]^{2-}$	$C_{44}H_{45}O_{25}Al$	500.1038	500.1016	4.4
$[2PB + Al^{3+} - 7H^{+}]^{2-}$	$C_{88}H_{95}O_{50}Al$	989.2359	989.2363	0.4
$[3PB + Al^{3+} - 8H^{+}]^{2-}$	$C_{132}H_{145}O_{75}Al$	1478.3679	1478.3765	5.8
$[3PB + Al^{3+} - 9H^{+}]^{3-}$	$C_{132}H_{144}O_{75}Al$	985.2429	985.2385	4.5
$[3PB + Al^{3+} - 10H^{+}]^{4-}$	$C_{132}H_{143}O_{75}Al$	738.6803	738.6790	1.8

[a]) Calculations carried out on website https://www.chemcalc.org/

Table 4-SI. Relative signal intensity (percentage of the free flavylium signal) with varying metal/pigment molar ratios.

		Relative signal intensity			
Ion	Exp. *m/z*	0	1/6	1/3	1
$[P2 - 3H^{+} + Fe^{3+}]^{+}$	1002.1731	0.01%	0.80%	0.90%	0.90%
$[P5 - 3H^{+} + Fe^{3+}]^{+}$	1208.2307	0.40%	1.1%	1.2%	4.1%
$[PB - 3H^{+} + Fe^{3+}]^{+}$	1032.1836	0.03%	0.77%	0.90%	0.87%

Table 5-SI. Competition between iron binding and water addition in a pH 4.24 acetate buffer a). Simultaneous kinetic analysis of the spectral changes at 530 nm (free pigment) and 670 nm (iron complexes). k'_h and k'_{-h}: apparent rate constants for hydration and dehydration (determined from control experiments in the absence of iron). k_f and k_d: rate constants for the formation and dissociation of the complexes. k_{autox}: rate constant for the autoxidation of the Fe^{2+} complex.

Pigment	PB	P3
k'_h (x10^{-3}, s^{-1})	33.1 (± 0.1)	82.4 (± 0.2)
k'_{-h} (x10^{-3}, s^{-1})	29.8 (± 0.2)	13.0 (± 0.1)
$k_f(Fe^{2+})$ (M^{-1} s^{-1})	680 (± 7)	346 (± 16)
$k_d(Fe^{2+})$ (x10^{-4}, s^{-1})	53 (± 1)	-
k_{autox} (x10^{-4}, s^{-1})	49.5 (± 0.3)	187 (± 9)
$k_f(Fe^{3+})$ (M^{-1} s^{-1}) b)	10^4	10^4
$k_d(Fe^{3+})$ (x10^{-3}, s^{-1})	91.9 (± 0.6)	211 (± 5)
ε (x10^3, M^{-1} cm^{-1})	Fe^{II}-PB (670 nm): 3.6 (± 0.1)<br>Fe^{III}-PB (670 nm): 24 c)	Fe^{III}-PB (530 nm): 10.9 (± 0.1)<br>Fe^{III}-PB (670 nm): 13.5 d)

a) Successive addition of pigment (40 µM) and Fe^{2+} (1 equiv.). The concentrations of free colored and colorless forms at the time of iron addition were estimated from the molar absorption coefficient of the free colored forms at 530 nm: 26.7x10^3 (Pigment B) and 14.9x10^3 (Pigment 3) M^{-1} cm^{-1}. b) Set constant (values from previous work with synthetic flavylium ions [20]). c) Deduced from the spectrum at pH 7 assuming total binding. d) Deduced from the spectrum at pH 8 assuming total binding), 14900 (free pigment, 530 nm).

Table 6-SI. Period of time for 25% loss in color or in total pigment (residual flavylium content assessed after acidification) at pH 7, 50°C.

	**PA**	**P1**	**P3**	**P4**	**PB**
t_{25} color (h), no metal added	0.02	0.15	0.18	1.4	0.29
t_{25} color (h), 0.6 equiv. Fe^{2+}	0.03	0.47	0.29	4.6	4.5
t_{25} pigment (h), no metal added	4.5	4.4	2.0	3.7	2.6
t_{25} pigment (h), 0.6 equiv. Fe^{2+}	0.12	1.4	1.9	5.2	5.5

t_{25} pigment (h), no metal added *vs* t_{25} pigment (h), 0.6 equiv. Fe^{2+}
